# PS-CARA: Context-Aware Resource Allocation Scheme for Mobile Public Safety Networks

**DOI:** 10.3390/s18051473

**Published:** 2018-05-08

**Authors:** Zeeshan Kaleem, Muhammad Zubair Khaliq, Ajmal Khan, Ishtiaq Ahmad, Trung Q. Duong

**Affiliations:** 1 Department of Electrical Engineering, COMSATS University, Wah Campus, Wah Cantonment 47040, Pakistan; zeeshankaleem@gmail.com (Z.K.); mrzubairkhaliq@gmail.com (M.Z.K.); 2 Department of Electrical Engineering, COMSATS University, Attock Campus, Attock 43600, Pakistan; drajmal@ciit-attock.edu.pk; 3 Department of Electronics Engineering, Inha University, Incheon 22212, Korea; ishtiaq001@gmail.com; 4 School of Electronics, Electrical Engineering and Computer Science, Queen’s University Belfast, University Rd, Belfast BT7 1NN, UK

**Keywords:** context-aware resource allocation, interference reduction, internet-of-things, public safety, 5G systems

## Abstract

The fifth-generation (5G) communications systems are expecting to support users with diverse quality-of-service (QoS) requirements. Beside these requirements, the task with utmost importance is to support the emergency communication services during natural or man-made disasters. Most of the conventional base stations are not properly functional during a disaster situation, so deployment of emergency base stations such as mobile personal cell (mPC) is crucial. An mPC having moving capability can move in the disaster area to provide emergency communication services. However, mPC deployment causes severe co-channel interference to the users in its vicinity. The problem in the existing resource allocation schemes is its support for static environment, that does not fit well for mPC. So, a resource allocation scheme for mPC users is desired that can dynamically allocate resources based on users’ location and its connection establishment priority. In this paper, we propose a public safety users priority-based context-aware resource allocation (PS-CARA) scheme for users sum-rate maximization in disaster environment. Simulations results demonstrate that the proposed PS-CARA scheme can increase the user average and edge rate around 10.3% and 32.8% , respectively because of context information availability and by prioritizing the public safety users. The simulation results ensure that call blocking probability is also reduced considerably under the PS-CARA scheme.

## 1. Introduction

The demand for ubiquitous availability of broadband data, low latency applications, and massive connectivity is compelling to instigate fifth-generation (5G) systems [1].

The industry and academia forecasted a 1000× increase in data traffic within the next decade. To meet these huge data traffic requirements, device-to-device (D2D) communications [2], ultra-dense small cells [3,4], and moving and flying networks [5,6,7,8] can be the the key enablers. The third generation partnership projects (3GPP) is evolving long term evolution-advanced (LTE-A) standards by introducing more features to achieve enormous data rate requirements. The number of users demands a massive data rate and reliable connectivity on the move while riding on buses, cars, and trains. To overcome these challenges, numerous European union (EU) projects such as EU FP7 and mobile and wireless communication enablers for the twenty-twenty information society (METIS) are focusing on meeting these demands by deploying moving networks [5,9,10,11]. The moving networks deployment brings the base station closer to the users, which in turn reduces the channel losses and latency, and increases the users data rate. The moving networks can be easily adopted in 5G systems due to its flexible and easily adoptable architecture. The moving networks can be the best candidate in disaster situations, where most of the base stations are not functional, to provide the emergency communications among users. Like numerous public safety standards such as trans-European trunked radio (TETRA) and project 25 (P25), 3GPP LTE is evolving existing standards to meet public safety application requirements.

Inspired by this, we presented 3GPP LTE-A based mobile personal cell (mPC) that follows users to meet their emergency data rate and latency demands. The mPC deployment can meet the public safety low latency and high priority requirements as it can expeditiously establish the communication link among the base station and public safety emergency users. However, mPC resource allocation poses some serious challenges because users frequent movements rapidly changes their location in disaster situations; this results in rapidly fluctuating channel conditions. Moreover, in disaster hit areas users also needs priority in connection establishment over the conventional users. So, the conventional static resource allocation schemes designed for low mobility or static environments are not suitable for varying channel conditions [12]. Therefore, mPC requires context-aware resource allocation schemes that can allocate resources among users using context information such as their location and connection establishment priority. Moreover, to efficiently manage the context-aware schemes, we need centralized information of the resource allocation.

**Figure 1:**
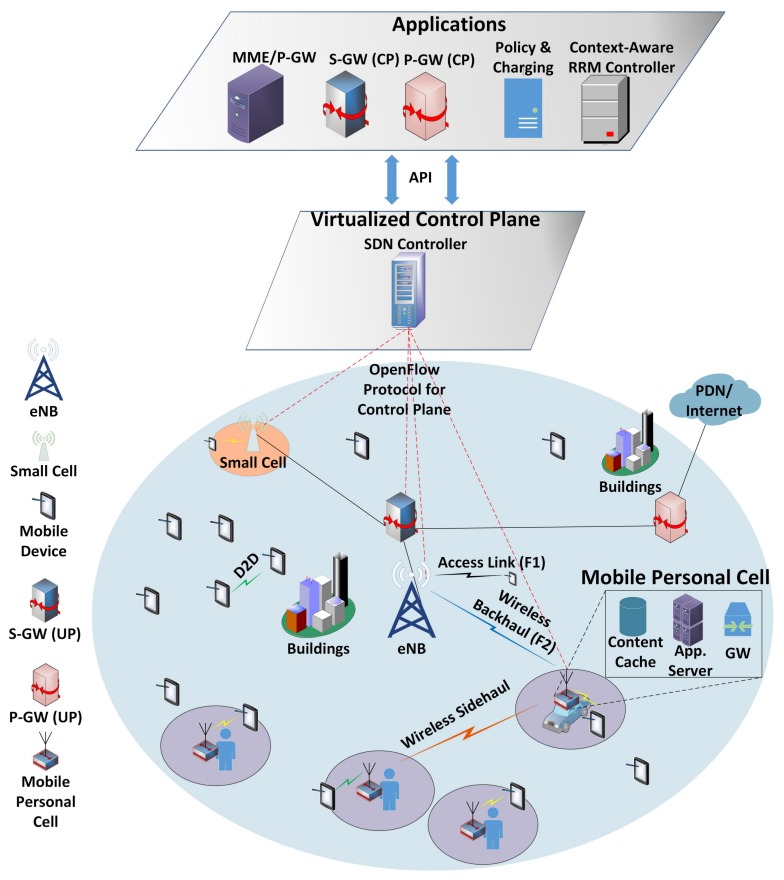
5G system architecture with mPC deployed to fulfill the users’ emergency requirements.

In this paper, we consider software-defined network (SDN) based architecture [13] which has the capability to centrally manage the mPC in its coverage area. The SDN architecture has the capability to centrally manage the necessary control activities such as network synchronization issues and centralized resource management of the disaster area. The SDN includes components like context-aware radio resource management (RRM) entity that centrally allocates the resources based on a situation and can easily centrally monitor the disaster hit areas. Moreover, it delivers central control signals to the public safety officers working in disaster areas. Furthermore, the user association [14] is performed based on public safety user priority. The use cases for deploying mPC in a 5G dense network is shown in Figure 1. An mPC deployed near the target areas can follow the target users, which can improve the throughput and quality of service (QoS).

However, mPC deployment has the issue of resource allocation because the rapid change in mPC position can result in severe co-channel interference. Moreover, it also requires mPC’s context information to efficiently allocate the resources, which restricts the utilization of the conventional resource allocation schemes discussed in [12], because those schemes statically allocate resources to users without considering the context information of mPC.

Most of the existing works on QoS-aware resource allocation such as QoS priority-based dynamic fractional frequency reuse (QoS-DFFR) [15] and QoS-based uplink power control [16,17] are discussed before to improve the sum-rate in heterogeneous networks with fixed small cells. Similarly, interference-aware power control [18], neighbor interference situation-aware power control [19], and load balancing with selective borrowing (LBSB) [20] also focused on optimizing the system sum-rate under the same deployment scenarios. Moreover, in [21] authors presented energy-efficient power allocation and wireless backhaul bandwidth allocation for small cell networks to enhance the throughput. The literature discussed above has deficiencies because it only focused on fixed small cells and no mobility was modeled.

## 2. Related Work

To meet the growing data rate demand for mobile data in urban areas, network densification is the best possible solution. The network densification can be achieved by deploying low-power small cells as they maximize the capacity by reusing the same frequency band. The small cells with moving capability can also fulfill the emergency communication requirement by deploying them in disaster hit areas.

Numerous interference management schemes related to moving networks are summarized in [22,23,24,25], but to the best of our knowledge these schemes manage the interference among moving networks by neglecting the context information. Hence, these schemes are not suitable for public safety situations where the user demands the emergency resources based on context information.

In [26], authors presented the context-aware resource allocation scheme by targeting energy consumption reduction. Similarly, the authors discuss mobile cloud computing based context-aware resource allocation in [27]. This scheme selects service providers which minimize the offloading time and maximize the mobile devices lifetime by satisfying deadline constraint. In [28], authors proposed a context information based resource allocation scheme. This scheme considers the energy level and communication quality as a part of user context information. The energy efficient algorithm based on the Gale–Shapley algorithm for context-aware resource allocation is presented in [29]. The presented schemes formulated the resource allocation problem as a joint partner selection and power allocation problem. Their simulation results indicate optimization in energy consumption at low complexity. However, this scheme was only proposed by targeting the static caching-enabled small cells.

Similarly, authors in [30] presented the context-aware resource allocation problem by jointly exploiting both the wireless channel and social context information of the users for D2D-enabled small cells. Their simulation results prove the importance of having both the social and channel conditions; that is the users with context-aware resource allocation can achieve the best possible results as compared with context-unaware approaches. The major problem in their work was that they ignored the mobility, and thus that cannot be adopted for mPC. Hence, none of the above discussed schemes are suitable for the public safety scenario as they ignored the mobility and most of them also did not consider the context information to allocate the resources among the users. This in turn results in more blocking and throughput reduction. To the best of our knowledge, no scheme is presented in the literature that jointly considers factors such as mobility, user priority for public safety situation, and the location information.

Contributions: Motivated by this, we propose a public safety users connection priority-based context-aware resource allocation (PS-CARA) scheme. The proposed PS-CARA scheme has the following differentiating characteristics: (1) It uses the context information (that is, public safety user priority and their location information) to allocate the resources among users. (2) Moreover, PS-CARA scheme dynamically adjusts the mPC spectrum access ratio η based on the number of public safety users, which in turn increase the users’ throughput. Subsequently, we further divided the available resource blocks into three parts, that is, for access links, backhaul links, and sidehaul links. (3) We also consider uplink (UL) power control scheme to reduce the interference among users. (4) Moreover, we consider two major factors for user association (a) priority indicator and (b) minimum pathloss factor [31], and hence it helps PS-CARA to reduce the call-blocking probability and interference among the users, and increase the public safety user throughput. We summarized the list of key mathematical symbols in Table 1.

## 3. System Model

We consider a two-tier HetNet UL scenario with underlayed mPC cellular network. A two-tier HetNet consists of a macrocell base stations (MBS) set M with M={1,....,M}, a set of K={1,....,K} mPCs that are randomly deployed in the coverage of each MBS, and a set of users U={1,....,U} with *N* and *L* are number of users attached per MBS and mPC, respectively. To clearly understand the system model, we plotted a single cell scenario with one BS and K mPC are deployed in its coverage area in Figure 1. We consider the SDN enabled architecture where control and data planes are partitioned to easily and centrally manage the public safety scenario. In SDN based core network architecture, the control plane (CP) functions are moved to an application layer, where we have core network components for control purposes such as packet data network gateway (P-GW), serving gateway (S-GW), mobility management entity (MME), policy and charging function (PCC), and context-aware radio resource management (CA-RRM). The functions of each component are briefly explained as: P-GW: it is mainly responsible for users’ IP address allocation, QoS provisioning, and charging based on the PCC rules. S-GW: the S-GW passes the IP packets of all users. The other functions of S-GW are to terminate the interface towards E-UTRAN, perform handovers among eNB, and to care about mobility interface to other networks. MME: it is the control node for LTE radio access. Some of the key responsibilities include user tracking, bearer activation and deactivation, P-GW and S-GW selection. PCC: the PCC consists of functions such as policy and charging rules function (PCRF), policy and charging enforcement function (PCEF), and online/offline charging system (OCS). The major functions of PCC include network control regarding service data flow detection, QoS control, and flow-based charging. Similarly, for user plane (UP) the functionality of the S-GW, P-GW is the same but will deal only with data and control will be managed centrally. The sidehaul links are considered to communicate among the mPC while backhaul link is to establish the link among the BS and mPC as shown in Figure 1.

**Figure 2:**
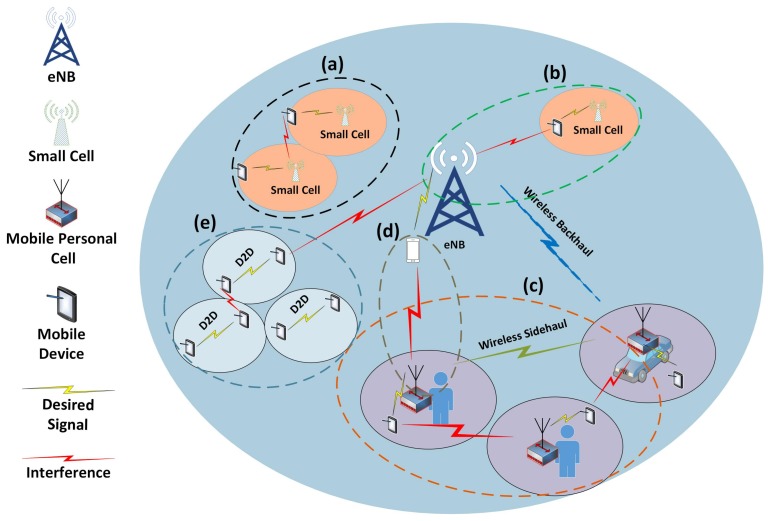
Illustration of various kinds of inter-cell interference after mPC deployment in a HetNet.

In this scenario, macro BS *m* transmits with a maximum power $P_{m}^{\mathrm{tx}}$, the mPC BS *k* transmits with maximum power of $P_{k}^{\mathrm{tx}}$, and the users *u* transmits with maximum power of $P_{u}^{\mathrm{tx}}$. We assume that each user *u* can associate with maximum one BS at a time. The total bandwidth (BW), *B*, is divided into *R* resource blocks (RBs) in the frequency domain. Since, there can be different types of small cells in the 5G systems, so the possible interference situations after deploying mPC in HetNet is shown in Figure 2. There are five different uplink interference scenarios in a HetNet, as shown in Figure 2(a–e). From Figure 2, scenario (a) depicts the uplink interference at the small cell base station from the neighbor small cell users reusing the same frequency band. Similarly, scenario (b) is the uplink interference at the MBS uplink communication from the neighbor small cell user. Moreover, in scenario (c) there is an uplink interference from the neighbor mPC user to the mPC base station. Furthermore, we also have an uplink interference from mPC user to MBS in scenario (d). Finally, in scenario (e) there is an uplink interference from the neighbor device-to-device (D2D) user to the MBS. From the mentioned scenarios, we only focus on scenario (c) and (d) where we have UL interference from mPUE → *m*-th BS and from MUE → *k*-th mPCs for backhaul, sidehaul, and access links. The major difference among the conventional small cells and mPC interference is the rapidly changing environment which in turn needs context-aware resource allocation schemes to counter this interference.

In the literature, numerous user association schemes are discussed. For instance, in [32] authors proposed the user association and power allocation scheme for mmWave networks. This scheme jointly considers load balancing, energy efficiency, QoS requirements, and energy harvesting constraints to model this problem. However, unfortunately, this scheme cannot be adopted for the public safety prioritized mobile scenario because they ignored the mobility and the PS user prioritization. Therefore, in this paper, we modeled the user-association problem as the public safety priority-based user association [14] that can be represented as

(1) $$\psi_{u,j}=\arg\max_{k\in \{1,2,\ldots .,K\}}\delta_{u,j}L_{u,j}^{\min},$$

where $L_{u,j}^{\min}$ is the minimum pathloss of user *u* from BS *j*, and that BS can be either macro BS (MBS) or mPC and $\delta_{u,j}\in \left\{0,1\right\}$ is the priority indicator.

Since all users u∈U are randomly distributed in the coverage area of MBS. Hence, there is a possibility that some users would exist at the cell edge, which in turn will cause high co-channel interference. Thus, to reduce interference, *fractional power control* (fpc) [33] is implemented for public safety (PS) and non-PS users transmissions, where user transmit power $P_{u}^{\mathrm{tx}}$ can be calculated as

(2) $$P_{u}^{\mathrm{fpc}}\left(dB\right)=\min\left\{P^{\max},P_{o}+\alpha L\right\},$$

where $P_{0}=P_{0}^{\mathrm{cell}}+P_{0}^{\mathrm{UE}}$ is the signal-to-interference-plus-noise-ratio (SINR) target control parameter, with $P_{0}^{\mathrm{cell}}$ and $P_{0}^{\mathrm{UE}}$ being the cell-specific and user specific parameters, respectively. These values can be selected within the specified ranges as $P_{0}^{\mathrm{cell}}=\left[-126,24\right]$ dBm and $P_{0}^{\mathrm{UE}}=\left[-8,7\right]$ dB. In addition, α∈[0,1] is the path loss compensation factor, which is selected as α=0.7 from the interval because it provides good coverage and better signal-to-interference-and-noise-ratio (SINR). The UL received signal from the *u*-th transmitting (tx) user (mPUE/MUE) to *k/m*-th receiving (rx) BS (mPC/MBS) is written as

(3) $$\begin{array}{c} y_{u\to k}^{\mathrm{rx}}\left(j,r\right)=h_{u\to k}\left(j,r\right)\sqrt{P_{u\to k}^{\mathrm{tx}}\left(j,r\right)}x_{u\to k}+\sum_{u=2,u\neq 1}^{U}\\ h_{u}\left(j,r\right)\sqrt{P_{u}^{\mathrm{tx}}\left(j,r\right)}x_{u}\left(j,r\right)+n_{u}. \end{array}$$

In (3), $x_{u\to k}$ is the transmitted data, $h_{u\to k}\left(j,r\right)$ is the channel gain between the u-th tx user and the *k*-th rx user during the *j*-th subframe and on the *r*-th RB. Furthermore, $P_{u\to k}^{\mathrm{tx}}\left(j,r\right)$ is the transmitted power of the *u*-th user to *k*-th BS, whereas $h_{u}\left(j,r\right)$ is the channel gain of the U−1 interfering users, and $n_{u}$ is the additive white Gaussian noise (AWGN) at the receiver with variance $\sigma^{2}$ for the u-th user. Similarly, (3) can be adopted to model the SH and BH received signals. Thus, user received SINR (γ) can be evaluated as

(4) $$\gamma_{u\to k}^{\mathrm{rx}}\left(j,r\right)=\frac{P_{u\to k}^{\mathrm{tx}}\left(j,r\right){\left|h_{u\to k}\left(j,r\right)\right|}^{2}}{\sum_{u=2,u\neq 1}^{U}{\left|h_{u}\left(j,r\right)\right|}^{2}P_{u}^{\mathrm{tx}}\left(j,r\right)+\sigma^{2}}.$$

The channel gain $h_{u}$ in (4) can be expressed as $h_{u}=FSL$, where *L*, *F*, and *S* are the pathloss, fading, and shadowing models, respectively. In this paper, *L* for user *u* connected to MBS and mPC can be evaluated by using Urban area path loss model and WINNER + B1 model [34], respectively. The urban area path loss model is considered to model the path loss between the outdoor MBS and outdoor user connected with MBS based on 3rd generation partner ship project (3GPP) recommendation [35]. To model the path loss between the user connected to the mPC and user, we consider WINNER + B1 model with line-of-sight (LOS) scenario as suggested in [34] for moving networks. Moreover, different values used in these path loss models are calculated based on different parameters such as base station and user antenna height, and carrier frequency [36]. The path loss models considered are

(5) $$L\left(dB\right)=\left\{\begin{array}{cc} 15.3+37.6\log10(d) & \text{Urban area model}\\ 35.4+22.7\log10(d) & \mathrm{WINNER}+B1, \end{array}\right.$$

where *d* is the distance from user *u* to BS. The fading *F* is calculated by using Ped-B model defined by International Telecommunication Union (ITU) [37]. The shadowing *S* follows the log-normal distribution, that is if any positive random variable X∈S is log-normally distributed, then their logarithm is normally distributed N(μ,σ).

To efficiently allocate the access link (AL) resources among users, mPC spectrum access ratio η∈{0,1} is introduced. This can dynamically adjust the allocated BW based on mPC load conditions [14]. That is, if the number of mPUEs are less in a system, then remaining BW is distributed among the non-PS users. By simulations, we found that η=0.4 and η=0.7 suits for non public safety and public safety scenarios, respectively. Moreover, two indicators are introduced in this paper: (1) the user association indicator $\beta_{u,m/k}\in \left\{0,1\right\}$ which indicates whether user *u* is associated with m/k-th BS and (2) the public safety priority indicator variable $\delta_{u}\in \left\{0,1\right\}$, where $\delta_{u}=0$ indicates that user *u* is non-public safety user and $\delta_{u}=1$ indicates it is a public safety user.

Since our target is to associate users based on the loading conditions of BSs. The total load on k-th MBS and m-th mPC is calculated as

(6) $$\begin{array}{cc} \rho_{k}^{\mathrm{PS}/\mathrm{NPS}}= & \sum_{u\in U}\beta_{u,k}r_{u}, \end{array}$$

(7) $$\begin{array}{cc} \rho_{m}^{\mathrm{PS}}= & \sum_{u\in U}\delta_{u}\beta_{u,m}r_{u}, \end{array}$$

(8) $$\begin{array}{cc} \rho_{m}^{\mathrm{NPS}}= & \sum_{u\in U}\beta_{u,m}r_{u}, \end{array}$$

where $r_{u}$ is the allocated RB to user *u*. Moreover, in (7) there is an additional $\delta_{u}$ parameter that allows only PS users with high priority to access the mPC spectrum. Based on these loads, the total achievable data rate for the users connected with MBS and mPC are evaluated as

(9) $$\begin{array}{cc} C_{k}^{\mathrm{NPS}}= & \rho_{k}^{\mathrm{PS}/\mathrm{NPS}}\left[R+\left(1-\eta \right)R\right]\sum_{u\in U}\left(1+\gamma_{u\to k}^{\mathrm{rx}}\left(j,r\right)\right), \end{array}$$

(10) $$\begin{array}{cc} C_{m}^{\mathrm{PS}}= & \rho_{m}^{\mathrm{PS}}R\eta \sum_{u\in U}\left(1+\gamma_{u\to k}^{\mathrm{rx}}\left(j,r\right)\right), \end{array}$$

(11) $$\begin{array}{cc} C_{m}^{\mathrm{NPS}}= & \rho_{m}^{\mathrm{NPS}}R\eta \sum_{u\in U}\left(1+\gamma_{u\to k}^{\mathrm{rx}}\left(j,r\right)\right), \end{array}$$

where in (9), the term (R+(1−η)R) represents that the MBS users (MUE) can access some RBs of mPC based on η. If enough free spectrum of mPC is available then η reduces, and non-PS users can also access the freely available mPC spectrum.

## 4. Problem Formulation

In this section, our objective is to maximize the system sum-rate of PS and non-PS users connected with either MBS or mPC by considering the SINR, transmit power, and priority constraints. Thus, we propose the sum-rate maximization problem (P1) as

(12a) $$\begin{array}{cc} P1:\max & \;\left(\sum_{j=1}^{\mathrm{maxTTI}}\left(C_{k,j}^{\mathrm{NPS}}+C_{m,j}^{\mathrm{NPS}}+C_{k,j}^{\mathrm{PS}}\right)\right) \end{array}$$

(12b) $$\begin{array}{cc} s.t. & \gamma_{u}^{\mathrm{rx}}\left(t\right)\geq \gamma_{th}, \end{array}$$

(12c) $$\begin{array}{c} \;\delta_{u}\in \left\{0,1\right\} \end{array}$$

(12d) $$\begin{array}{c} \;P_{u}^{\mathrm{fpc}}\leq P_{u}^{\mathrm{tx}}\left(t\right)\leq P_{u}^{\max}. \end{array}$$

Since we are also dealing with connection level priority instead of a service level priority model, call blocking probability (CBP) is a good performance metric to judge the quality of the system. The CBP measures the call admission rejection probability because of the limited available resources. The CBP (ξ) of a user *u* trying to associate with BS can be given as

(13) $$\begin{array}{c} \xi_{u}=1-\frac{\sum_{j=1}^{\mathrm{maxTTI}}S_{j}}{U}, \end{array}$$

where $S_{j}$ is the number of user scheduled during the j-th subframe and *U* is the total number of user sending the admission request. In this paper, we also minimize the CBP of the users associated with the BS, by considering the user association and priority constraints. Thus, CBP minimization problem (P2) can be evaluated as

(14a) $$\begin{array}{cc} P2:\min & \;\xi_{u} \end{array}$$

(14b) $$\begin{array}{cc} s.t. & \gamma_{u}^{\mathrm{rx}}\left(t\right)\geq \gamma_{th}, \end{array}$$

(14c) $$\begin{array}{c} \;\delta_{u}\in \left\{0,1\right\} \end{array}$$

(14d) $$\begin{array}{c} \;\beta_{u}\in \left\{0,1\right\} \end{array}$$

(14e) $$\begin{array}{c} \;P_{u}^{\mathrm{fpc}}\leq P_{u}^{\mathrm{tx}}\left(t\right)\leq P_{u}^{\max}. \end{array}$$

The optimization problems discussed here are NP-hard because it has the possibility of allocating a huge number of RB combinations for cellular and mPC users. The maximum number of possible links in this scenario are ${\left(M\times N+K\times L\right)}^{RB}$ [38], where RB are the total number of available resource blocks to establish communication among all users.

Hence, the solutions to these problems are not feasible and only a sub-optimal solution can be achieved. To solve these problems, we propose a PS-CARA scheme that heuristically solves the constructed optimization problem (P1) in (12) and (P2) in (14) by system-level simulations. By these simulations we found the sub-optimal solution to these problems. The proposed PS-CARA scheme’s major steps are summarized in the next section.

## 5. Proposed PS-CARA Scheme for mPC

Deployment: We propose PS-CARA scheme under the SDN-based cloud network architecture because of its capability to centrally manage all the base stations. The number of mPC required per macro BS varies according to the deployment situation or scenario. That is, in the PS scenario a higher number of mPCs are desired to meet the user requirements. Therefore, more mPCs are deployed in PS scenario and the resources are allocated by considering the context information of the users.

**Figure 3:**
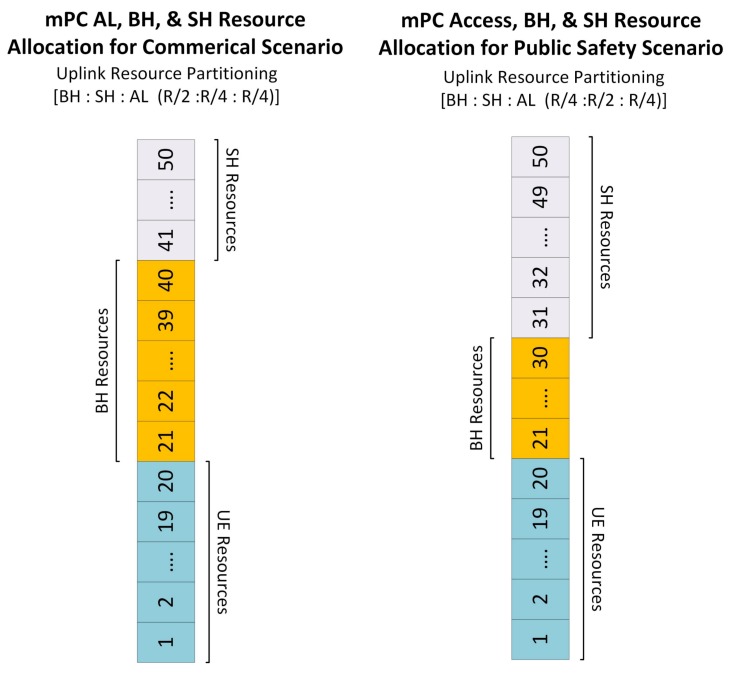
Dynamic resource allocation for access, backhaul, and sidehaul links.

BH, SH, and AL resource allocation: In the proposed scheme, mPUE and MUE are connected to mPC and MBS, respectively via AL, whereas mPCs are connected to MBS via BH links. Similarly, mPC is connected with neighbor mPC by using SH links. All these links are using the orthogonal frequency band in order to avoid interference. mPC can switch to use different BH and SH modes to allocate different ratio of resources for BH and SH depending on the nature of deployment situation. That is, more BH resources can be reserved for non-PS deployment and less for PS scenario. For example, the system with 10 MHz BW and 50 RBs in non-PS scenario, has RB distribution ratio of BH:SH:AL=R/2:R/4:R/4, whereas for PS scenario, this ratio can be BH:SH:AL=(R/4:R/2:R/4). The reason for this dynamical variation of RBs is the deployment situation, because in non-PS scenario, there is a lower possibility of SH communication between the mPCs. While in the PS situation, due to multi-hop communication between mPC, there is more demand of SH communication, and hence more SH resources are required. Thus, in the proposed PS-CARA scheme, these resources are dynamically adjusted, and one of the possible combinations is shown in Figure 3.

mPC call admission control (CAC): In LTE system, CAC determines whether to accept or reject a new call based on the availability of sufficient network resources to guarantee the QoS parameters without affecting the existing calls. Since CAC is the connection provisioning module in mPC for the user initially accessing the system. Therefore, CAC is capable of identifying the call type at any moment. Hence, the number of PS users are decided based on the connection request information. However, if there is a large number of PS users sending the connection request to mPC, then mPC will switch to PS mode from the non-PS mode and start assigning resources based on PS scenario deployment configuration.

Context-information collection: We use the context information by considering the PS priority of the users to dynamically schedule and allocate the resources. There are two types of context information (1) location information which is decided based on received SINR, that is if $\gamma^{\mathrm{rx}}<\gamma^{\mathrm{th}}$, user is located at cell-edge and vice versa, (2) the number of PS users sending the emergency connection request is decided by CAC. The users are prioritized by considering the connection priority defined in Table 2. In this paper, we modeled the PS scenario where PS users are greater than the non-PS users, and generally we divided the users into two broad categories, that is PS users and commercial non-PS users. Since our interest is to check the performance of PS scenario, we deploy a greater number of PS users as compared with non-PS users. The priority of the users is decided by the priority indicator variable δ, that is when δ=1, the user has high priority and vice versa.

**Figure 4:**
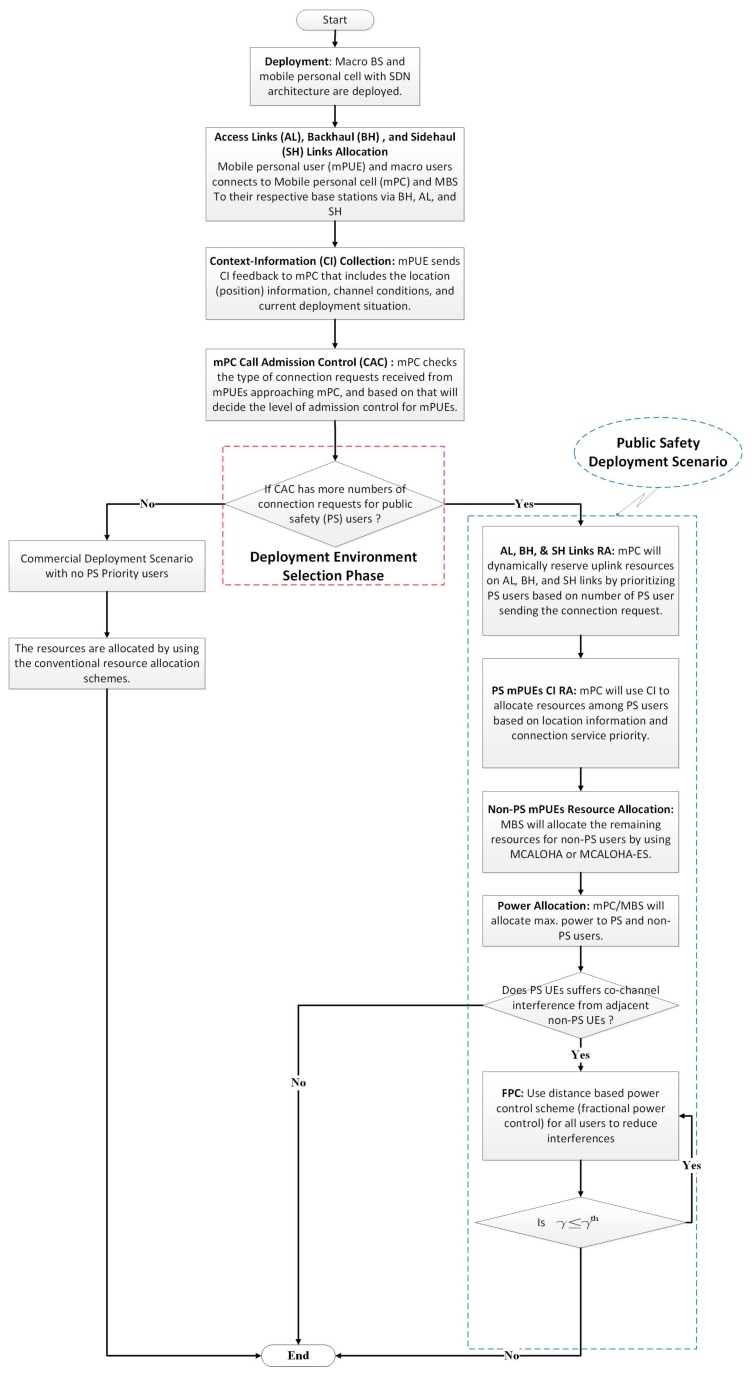
Propose PS-CARA scheme for mPC in public safety scenario.

The main steps in the proposed PS-CARA scheme are shown in Figure 4. The important steps of the proposed PS-CARA scheme are also described here by providing brief details as

**PS and non-PS user resource allocation**: During this phase, firstly, all resources are reserved for PS users with high connection priority; then, based on round robin scheduling these resources are distributed among the high-priority users to maximize their sum rate. For low priority non-PS users, the remaining resources are allocated randomly between the non-PS users by using multi-channel slotted ALOHOA (MCALOHA) protocol. To improve the efficiency of resource allocation for non-PS users, MCALOHA is combined with energy sensing (MCALOHA-ES) if interference is very high [39]. In MCALOHA-ES resource allocation, user *u* considers received energy levels to allocate RBs among non-PS users.

**PS and non-PS users fpc implementation**: Initially, all users are deployed in a system by allocating full transmission power. This may result in high interference from the neighboring users. To control this situation, fpc is implemented that allocates appropriate power to users by considering their locations, which in turn will reduce the interference.

Before implementation of the PS-CARA scheme, it is important to analyze the complexity to prove its suitability. The PS-CARA scheme summarized in Figure 4, where initially the proposed scheme allocates the dedicated resource to AL, SH, and AL links. After the environment selection phase, we have iterations that decide which two users share the current RB.

The complexity of the proposed PS-CARA scheme is reduced as compared to the existing schemes because we separately allocate resources for PS and non-PS users, and hence the resource selection competition is separately among PS and non-PS users. Therefore, the proposed PS-CARA is a sub-optimal scheme that requires a modest complexity, and it has a linear relationship with the number of RBs as compared to the huge number of possible combinations regarding the NP-hard problem. Since we have a distributed network and each node decision is independent of the other node, this results in less system complexity.

## 6. Simulation Results and Discussions

The performance of the proposed PS-CARA scheme is evaluated by upgrading the system-level simulator designed in MATLAB [40]. This simulator is designed by taking care of the assumptions given for the PS scenario [34]. The key assumptions considered during simulations are: (1) user can associate to one BS at one time, that is either with MBS or mPC, (2) only uplink interference scenario is modeled, (3) frequency division duplexing (FDD) is considered, (4) urban macro scenario is considered because this scenario is compulsory for both general purpose scenario and public safety scenario [34], and (5) all users and mPC are deployed outside only in the coverage area of MBS. In Table 3, we summarized the parameters used to perform the simulations.

We compare the performance of the proposed PS-CARA scheme with the conventional static resource allocation (C-SRA) scheme, which ignores the context information during resource allocation.

### 6.1. Random-Walk Mobility Model for mPC

**Figure 5:**
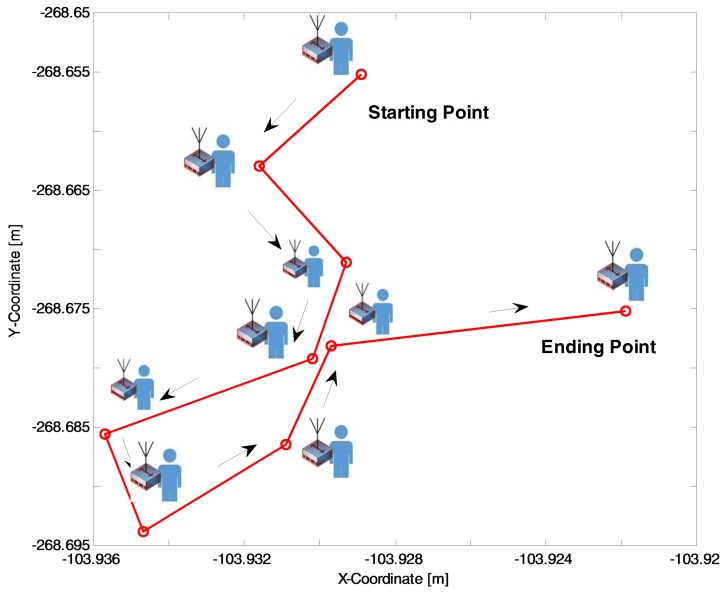
mPC traveling pattern in public safety deployed area.

The mobile mPCs are modeled in simulations by considering the random-walk mobility model. In Figure 5, we plotted mPC moving path by using random-walk mobility model. We considered this model because it is suitable for low speed mobile networks. The other reason for opting for the random-based mobility model is because here the mPC can move randomly without any restrictions. To be more specific, each mPC speed, destination, and direction is chosen randomly without relating other mPC. Hence, the random-walk mobility model is considered to model the movement in the public safety scenario.

### 6.2. Effect of Varying SINR Target Control $\left(P_{0}\right)$ and Path Loss Compensation Factors (α) on User Transmit Power

The performance of the PS-CARA scheme is considerably improved as compared to the C-SRA scheme because of implementing fraction power control scheme. The selection of power control parameter is very important to provide the better performance, and its selection varies for each environment. The main parameters of concern in fractional power control are; SINR target control $\left(P_{0}\right)$ parameter and path loss compensation factor (α). The improper selection of these parameters can result in high interference and coverage degradation which in turn will degrade the system sum-rate. Thus, we need to select the value which gives us maximum coverage but at the cost of less interference.

**Figure 6:**
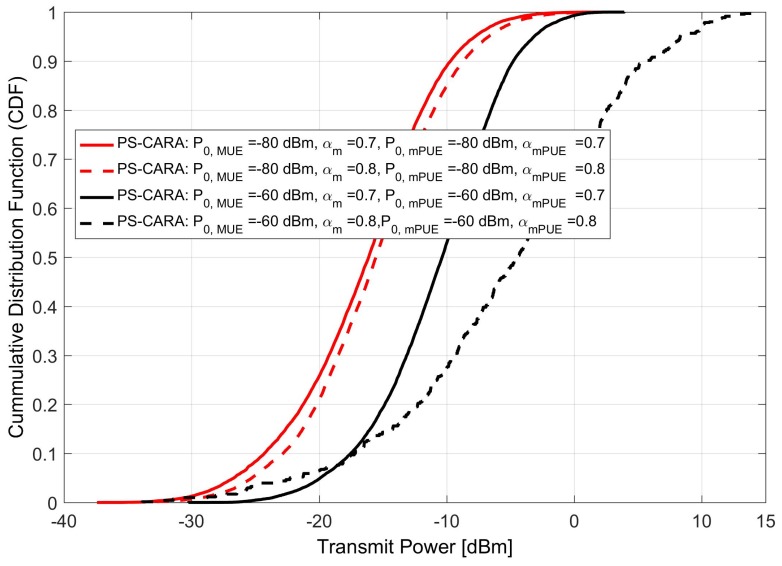
User transmit power under the proposed PS-CARA scheme for various $\left(P_{0}\right)$ and (α).

To select a proper value of $\left\{P_{0},\alpha \right\}$, firstly we checked the user transmit power at lower values $\left\{P_{0},\alpha \right\}=\left\{-80\;\mathrm{dBm},0.7\right\}$ for both MUE and mPUE. Secondly, we keep the $P_{0}$ constant and changed α=0.8. After comparing these two cases, we found that by increasing α=0.7 → α=0.8, the users need to transmit with more power to compensate the path loss. This will in turn increase interference as more users will start transmitting with relatively high power as shown in Figure 6, and hence degrades the system sum-rate. A similar trend is noticed when user transmit power is plotted for the values $\left\{P_{0},\alpha \right\}=\left\{-60\;\mathrm{dBm},0.7\right\}$ and $\left\{P_{0},\alpha \right\}=\left\{-60\;\mathrm{dBm},0.8\right\}$. Thus, based on these results we selected $\left\{P_{0},\alpha \right\}=\left\{-80\;\mathrm{dBm},0.7\right\}$ for the simulations as it will result in the best sum-rate performance due to less interference.

### 6.3. Users Receive Interference under the PS-CARA Scheme

The performance of the proposed PS-CARA scheme is tested under the PS scenario. The PS-CARA scheme is verified for three possible cases: (1) PS-CARA: PS and Non-PS users without power control, (2) PS-CARA: with all users using power control, and (3) PS-CARA: PS UE without power control and Non-PS UE with power control. The reason for simulating these cases is to check the suitability of the PS-CARA scheme under different power control situations, and then to find the best suitable case.

**Figure 7:**
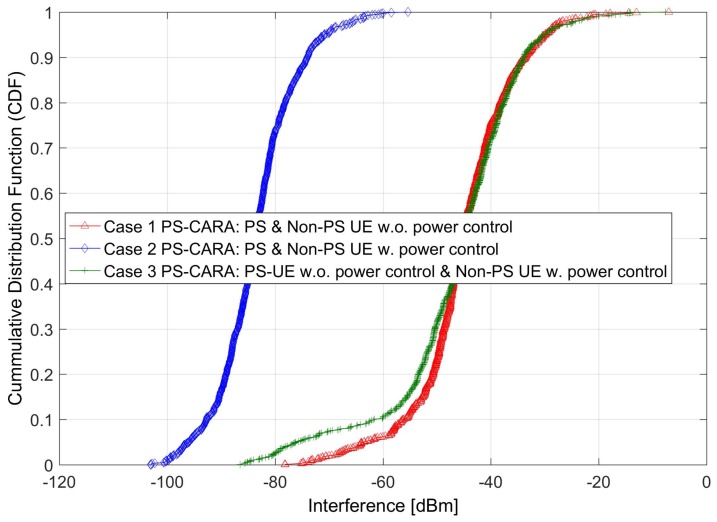
User received interference under the proposed PS-CARA scheme.

The simulation results depict that the proposed PS-CARA scheme results in very low interference for case 2, where power control is applied by considering the location and deployment situation of PS and non-PS users. The results in Figure 7 clearly demonstrate that by applying PS-CARA with power control, around 70% of total users lie in between the low interference range of −100 dBm to −80 dBm, whereas for the PS-CARA without the power control scheme, only 3% of total users are in the specified low-interference range.

**Figure 8:**
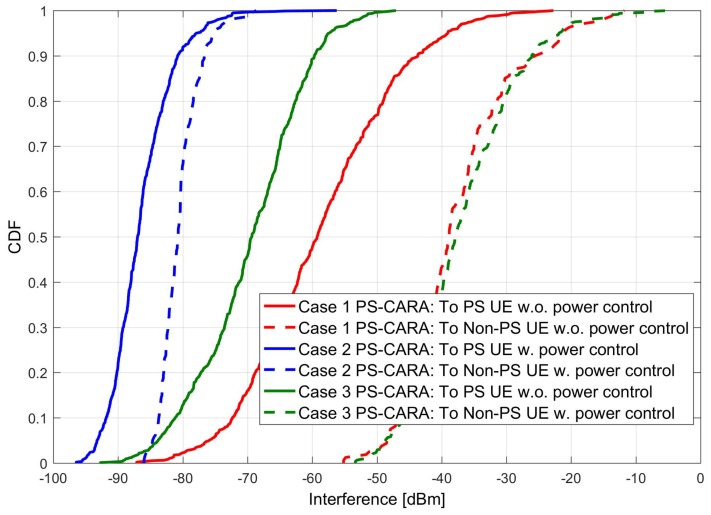
PS and non-PS users’ individual interference under the proposed PS-CARA scheme.

Furthermore, we individually plotted the effect of interference on PS and non-PS users for the three cases described above. From the simulation results shown in Figure 8 we can clearly notice that the case 2 PS-CARA, with all users using power control, is the best possible solution to reduce the interference. Hence, based on these results, we conclude that PS-CARA with power control is the best possible option in the public safety scenario as it reduces the interference remarkably.

### 6.4. Users Throughput for PS-CARA Scheme

The performance of the proposed PS-CARA scheme is compared with respect to the average (50%) and edge throughput (5%). The simulation results shown in Table 4 clearly demonstrate the effect of adding the mPC to the conventional MBS system. The simulation results clearly demonstrate that by deploying mPC, the average and edge throughput increases by around 10.3% and 32.8%, respectively. Moreover, we also notice that by deploying a greater number of mPC per MBS, the average and edge throughput of the system increases. The average and edge throughput increases by deploying a greater number of mPC because it reduces the call blocking probability because of having additional resources for the emergency users. However, there is a chance of an increase in co-channel interference, but that is countered by using the PS-CARA scheme which has the capability of reducing the interference because of introducing power control.

### 6.5. Call Blocking Probability

**Figure 9:**
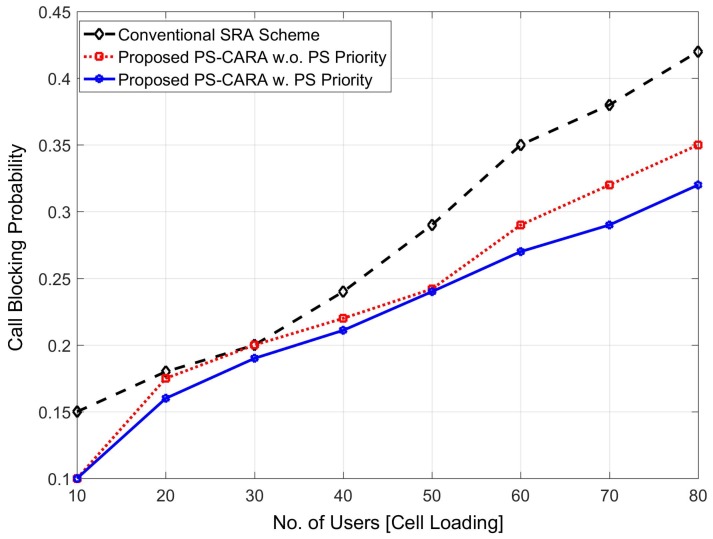
Call blocking probability of the proposed PS-CARA scheme.

The proposed PS-CARA scheme also reduces the call blocking probability of the users as compared with the existing C-SRA scheme. The users call blocking probability decreases because of deploying a greater number of mPC in the public safety areas. This in turn reduces the traffic congestion which occurs during the public safety scenario because a higher number of users access the limited spectrum of the MBS at the same time. We compare the simulation results in three different aspects: (1) using only C-SRA scheme where we randomly allocate the resources among users, (2) proposed PS-CARA scheme without giving priority to public safety users during resource allocation, (3) proposed PS-CARA scheme with public safety priority, where public safety users have been given high priority during resource allocation. The simulation results depicted in Figure 9 show that the proposed PS-CARA scheme with public safety priority outperforms all the other cases without giving priority. Hence, by giving priority to PS users the CBP reduces because the connection and resources are provided to users on a priority basis, which in turn results in less congestion for the priority users.

## 7. Conclusions

This paper presents the public safety priority-based context-aware resource allocation (PS-CARA) scheme for interference reduction in the PS-LTE system. This scheme takes care of the context-information and mPC mobility while allocating resources among users. From the simulation results, we found that PS-CARA with power control is the best possible solution for PS scenario as we have around 67% less interference as compared to PS-CARA without using power control. Moreover, we also found that by giving priority to the PS users, the call blocking is reduced significantly as compared with the C-SRA scheme. Similarly, we notice that by deploying a greater number of mPC in PS scenario, we can increase the mean and edge throughput of the users. In the future, we have a plan to check the performance of the PS-CARA scheme by introducing the energy consumption constraint because energy consumption is one of the important constraints in the PS scenario.

## Figures and Tables

**Table 1:** Table of symbols for PS-CARA scheme.

Symbol	Definition
M	Set of macrocell base stations
K	Set of mobile personal cell base stations
U	Set of users involved in communication
$P_{m}^{\mathrm{tx}}$	*m*-th macro base station transmit power
$P_{k}^{\mathrm{tx}}$	*k*-th mobile personal cell transmit power
*B*	Bandwidth
*R*	Resource blocks
$\psi_{u,j}$	User association indicator for user *u* associated with *j*-th base station
*L*, *F*, *S*	Pathloss, fading, shadowing, respectively
$P_{o}$	Signal-to-noise-ratio (SINR) target control parameter
α	Pathloss compensation factor
η	Mobile personal cell spectrum access ratio
δ	Priority indicator
ρ	Load distribution
ξ	Call blocking probability
γ	SINR

**Table 2:** Users’ priority table for PS LTE system.

User Priority	User Identification	Traffic Class	Barring	Establishment Cause
PS First Responders	PS Emergency; PS1 to PS5	12–14	Barring for Special	High Priority Access
Commercial User Emergency	Commercial User Emergency	10	Barring for Special	Emergency
Commercial User Non-Emergency	Commercial User Non-Emergency	0–9	Low Barring Factor	Mobile Originating

**Table 3:** Simulation parameters.

Parameters	Values
Layout	Hexagonal with 7 Cell Sites (3 cells per site), Urban Macro
mPC/MBS	4
Carrier Frequency	2 GHz
Bandwidth, RB	10 MHz, 50
Inter-site Distance	500 m
UL Resource Partitioning (BH:SH:AL)	PS Scenario (R/4:R/2:R/4), Non-PS Scenario (R/2:R/4:R/4)
Antenna Configurations	1×1
Mobility Pattern	Random Walking Model
UE/mPC Speed	3 Km/h
BS/UE Transmission Power	MBS: 43 dBm, mPC: 30 dBm, UE: 23 dBm
$P_{0}$, α	−80 dBm, 0.7
No. of UEs	8/MBS, 2/mPC (PS UE: 60%, Non-PS UE: 40%)
Schedulers	Round Robin (PS UEs), MCALOHA/MCALOHA-ES (Others)
Pathloss Models	Urban (MBS), WINNER + B1 (mPC)
Fading Model	PedB
Priority Modeling	3GPP Access Class Barring Models
Thermal Noise	−174 dBm/Hz
Traffic Models	Full Buffer
Simulation Time	20 Drops, 500 Subframes

**Table 4:** Average and edge throughput comparison by adding mPC in conventional MBS.

MBS/Cell	mPC/MBS	Average (50%) Throughput (bits/s/Hz)					Edge (5%) Throughput (bits/s/Hz)				
		Total	MBS	mPC	mPC BH	mPC SH	Total	MBS	mPC	mPC BH	mPC SH
1	0	2.058	2.058	0	0	0	0.444	0.444	0	0	0
1	4	2.251	2.049	5.283	3.354	3.857	0.447	0.442	1.263	0.847	1.160
1	10	2.310	2.055	3.839	2.518	2.642	0.455	0.443	0.693	0.490	0.599
1	20	2.270	2.058	2.9061	1.983	1.845	0.590	0.446	0.960	0.431	0.155
